# Protein network analyses of pulmonary endothelial cells in chronic thromboembolic pulmonary hypertension

**DOI:** 10.1038/s41598-021-85004-z

**Published:** 2021-03-10

**Authors:** Sarath Babu Nukala, Olga Tura-Ceide, Giancarlo Aldini, Valérie F. E. D. Smolders, Isabel Blanco, Victor I. Peinado, Manuel Castellà, Joan Albert Barberà, Alessandra Altomare, Giovanna Baron, Marina Carini, Marta Cascante, Alfonsina D’Amato

**Affiliations:** 1 Department of Pharmaceutical Sciences, Università Degli Studi Di Milano, 20133 Milan, Italy; 2 Department of Biochemistry and Molecular Biomedicine and Institute of Biomedicine (IBUB), Faculty of Biology, University of Barcelona, Barcelona, Spain; 3 Department of Pulmonary Medicine, Hospital Clínic-Institut D’Investigacions Biomèdiques August Pi I Sunyer (IDIBAPS), University of Barcelona, Barcelona, Spain; 4 Centro de Investigación Biomédica en Red de Enfermedades Respiratorias (CIBERES), Instituto de Salud Carlos III (ISCIII), CB06/06/0011 Madrid, Spain; 5 Department of Pulmonary Medicine, Dr. Josep Trueta University Hospital de Girona, Santa Caterina Hospital de Salt and the Girona Biomedical Research Institut (IDIBGI), Girona, Spain; 6 Department of Cardiovascular Surgery, Institut Clínic del Tòrax, Hospital Clínic, University of Barcelona, Barcelona, Spain; 7 Centro de Investigación Biomédica en Red de Enfermedades Hepáticas y Digestivas (CIBEREHD)- CB17/04/00023 and Metabolomics Node At INB-Bioinfarmatics Platform, Instituto de Salud Carlos III (ISCIII), Madrid, Spain; 8 Department of Pharmacology, The University of Illinois College of Medicine, 909 S Wolcott Ave, COMRB 4097, Chicago, IL 60612 USA

**Keywords:** Proteomics, Cardiovascular diseases, Mass spectrometry

## Abstract

Chronic thromboembolic pulmonary hypertension (CTEPH) is a vascular disease characterized by the presence of organized thromboembolic material in pulmonary arteries leading to increased vascular resistance, heart failure and death. Dysfunction of endothelial cells is involved in CTEPH. The present study describes for the first time the molecular processes underlying endothelial dysfunction in the development of the CTEPH. The advanced analytical approach and the protein network analyses of patient derived CTEPH endothelial cells allowed the quantitation of 3258 proteins. The 673 differentially regulated proteins were associated with functional and disease protein network modules. The protein network analyses resulted in the characterization of dysregulated pathways associated with endothelial dysfunction, such as mitochondrial dysfunction, oxidative phosphorylation, sirtuin signaling, inflammatory response, oxidative stress and fatty acid metabolism related pathways. In addition, the quantification of advanced oxidation protein products, total protein carbonyl content, and intracellular reactive oxygen species resulted increased attesting the dysregulation of oxidative stress response. In conclusion this is the first quantitative study to highlight the involvement of endothelial dysfunction in CTEPH using patient samples and by network medicine approach.

## Introduction

Chronic thromboembolic pulmonary hypertension (CTEPH) is a major complication of pulmonary embolism, characterized by an abnormal increase of pulmonary artery pressure due to the presence of organised thromboembolic material in pulmonary arteries that has not resolved after a period of appropriate anticoagulant therapy^1^. The progressive increase of pulmonary vascular resistance in CTEPH may ultimately lead to right ventricular failure and death^1^ . CTEPH is a dual vascular disorder, that combines occluded pulmonary vessels and distal arteriopathy in non-occluded vessels. Inflammation, autoimmunity, and angiogenesis may also occur^2,3^. The mechanisms underlying the persistence of thrombotic lesions and distal arteriopathy are poorly understood. The vascular endothelium, constituted by a thin layer of endothelial cells (ECs), is at the interface between circulating blood and the vessel wall. It regulates homeostasis, platelet activity, leukocyte adhesion and thrombosis^4,5^. The endothelium comprises adherents and tight junctions and expresses proteins, such as VE-cadherin in association with a-, b-, and g-catenins; platelet endothelial cell adhesion molecule 1 (PECAM-1); cortical actin filaments linked with zonula occludens-1 (ZO-1), vinculin and α-actinin, involved in actin filament network. Oxidative stress causes phosphorylation and reorganization of occludin and PECAM-1, reduces levels of vascular endothelium catenins and actin-binding proteins, resulting in disruption of the tight and adherent junctions^6^.

Endothelial dysfunction plays a fundamental role in promoting thrombus formation and pulmonary vascular remodeling. In pathological conditions, the loss of endothelium homeostatic function induces the disorganization of the vascular wall and the consequent development of vascular lesions. E-selectin, intercellular adhesion molecule (ICAM)–1, tissue factor, and vascular endothelial growth factor (VEGF) receptors^7^ are specific markers of endothelium dysfunction. In addition Kv channels and the Ca^2+^ signal are dysregulated in the development of pulmonary vascular remodelling in patients with idiopathic pulmonary arterial hypertension (IPAH)^8^. Pulmonary endarterectomy (PEA) is the treatment of choice in CTEPH. The intravascular thrombotic material along with the adhered fibrotic endothelial layer is surgically removed. As a result, pulmonary hemodynamics improve dramatically, with a complete resolution of pulmonary hypertension in most cases^9–11^. The location occlusive lesions in distal vessels in several patients precludes their surgical removal. In these cases, pharmacological treatment and/or balloon pulmonary angioplasty (BPA) are potential therapeutic alternatives^12^.

The physiological mechanisms underlying CTEPH are still unknown and the present study of in depth description of patient endothelium’s phenotypes may help to get an early diagnosis and better prognosis of CTEPH disease. Label free quantitative proteomics approach and network analysis of differentially regulated proteins in patient-derived cells provide a unique opportunity to investigate the molecular mechanisms of the disease.

## Results

### Differentially regulated protein pathways in CTEPH

**Figure 1:**
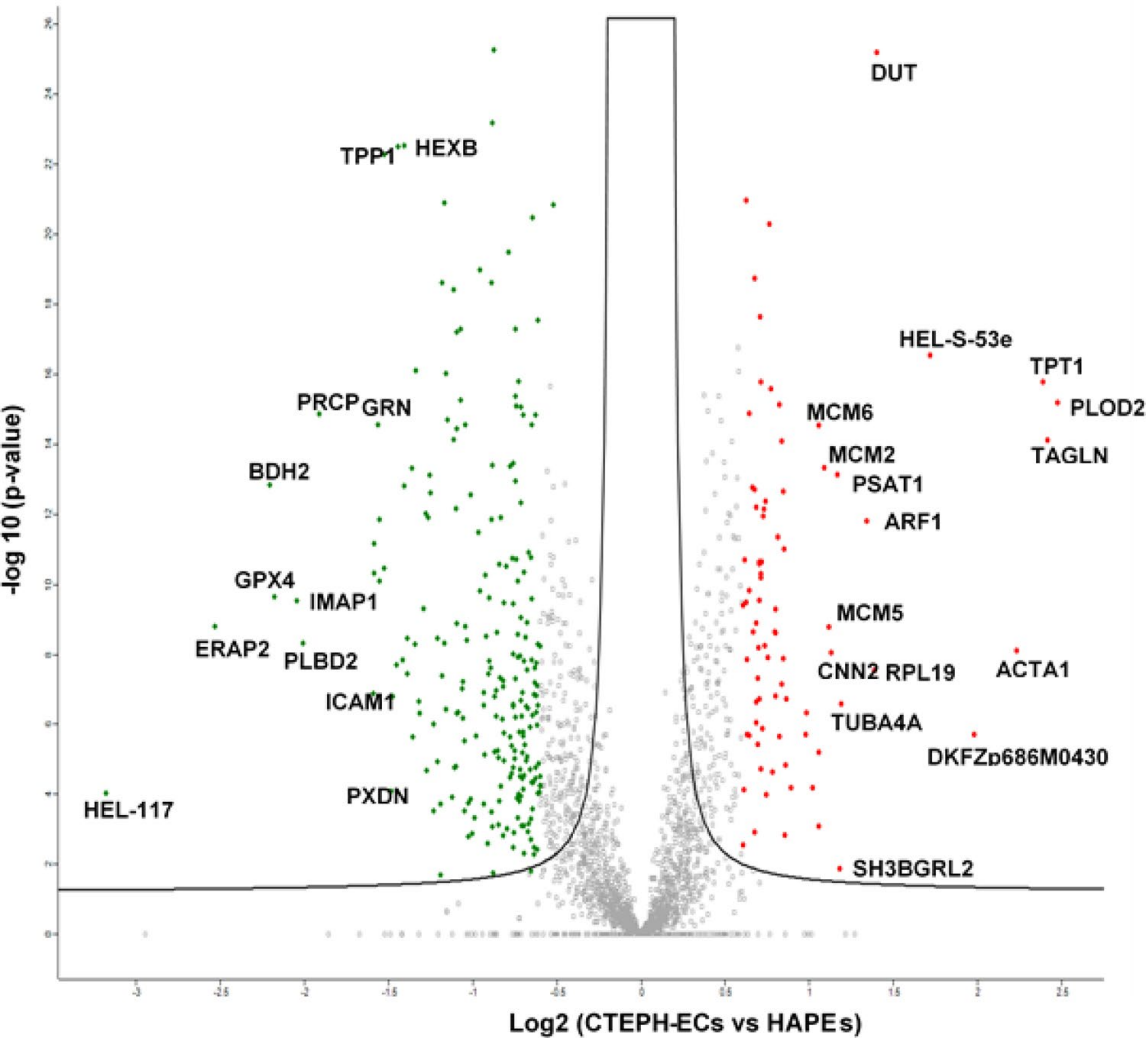
Distribution of altered proteins in CTEPH-ECs cells. In the above scatter plot, X-axis represents log2 ratio and y-axis represent − log p value of significantly differentially regulated proteins. Green colour symbolizes down-regulation, red colour symbolizes upregulation.

To characterize the potential molecular mechanisms underlying endothelial dysfunction in the development of the CTEPH, patient-derived CTEPH-ECs and healthy Human Pulmonary Artery Endothelial Cells (HPAECs) were analyzed by label free quantitative proteomics approach. 3258 proteins and 27,678 unique peptides were identified and quantified. 673 proteins were differentially regulated of which 82 proteins were overexpressed, and 232 proteins resulted down-regulated in the CTEPH-ECs compared to the HPAEs group, with a ratio’s threshold of 1.5 (Fig. 1, Table 1, Suppl. Table 1). Procollagen-lysine, 2-oxoglutarate 5-dioxygenase 2 (PLOD2, ratio = 5.50) and Transgelin (TGLN, ratio = 5.28), proteins involved in the cross-linking respectively of collagen and actin, resulted highly overexpressed, indicating the presence of fibrosis in CTEPH ECs^13,14^.

**Table 1:** List of protein differentially regulated in CTEPH patients versus controls.

Protein Accession numbers	Protein names	Gene names	log2 (ratio)
O00469	Procollagen-lysine,2-oxoglutarate 5-dioxygenase 2	PLOD2	2.47
Q5U0D2	Transgelin	TAGLN	2.41
A0A0P1J1R0	Translationally-controlled tumor protein	TPT1	2.38
P68133	Actin, alpha skeletal muscle;	ACTA1	2.23
V9HW83	Retinal dehydrogenase 1	HEL-S-53e	1.71
H0YNW5	Deoxyuridine 5-triphosphate nucleotidohydrolase, mitochondrial	DUT	1.40
J3QR09	Ribosomal protein L19;	RPL19	1.38
Q5ISS1	ADP-ribosylation factor 1;	ARF1	1.34
P68366	Tubulin alpha-4A chain	TUBA4A	1.18
Q9UJC5	SH3 domain-binding glutamic acid-rich-like protein 2	SH3BGRL2	1.17
A0A024R222	Phosphoserine aminotransferase	PSAT1	1.16
B4DDF4	Calponin;	CNN2	1.13
B1AHB1	DNA helicase;DNA replication licensing factor MCM5	MCM5	1.11
B3KXZ4	DNA helicase;DNA replication licensing factor MCM2	MCM2	1.08
Q14566	DNA replication licensing factor MCM6	MCM6	1.05
Q8WX93	Palladin	PALLD	1.05
O95433	Activator of 90 kDa heat shock protein ATPase homolog 1	AHSA1	1.05
K7ELC2	40S ribosomal protein S15	RPS15	1.02
Q8NG19	Collagen alpha-1(XVIII) chain;Endostatin	COL18A1	-1.00
L7UUZ7	Integrin beta	ITGB3	-1.01
A0A0S2Z3L0	Electron transfer flavoprotein subunit alpha, mitochondrial	ETFA	-1.01
Q53YE7	Amine oxidase [flavin-containing] A	MAOA	-1.02
B3KNK4	Phosphatidate cytidylyltransferase	CDS2	-1.03
Q8WYJ5	Histidine triad nucleotide-binding protein 2, mitochondrial	HINT2	-1.03
B4E2S7	Lysosome-associated membrane glycoprotein 2	LAMP2	-1.04
A0A024R8T3	Acyl-coenzyme A oxidase;	ACOX1	-1.04
B4DEH0	39S ribosomal protein L10, mitochondrial	MRPL10	-1.05
A0A0S2Z5A5	Septin-9	43,352	-1.05
Q96N83	Podocalyxin	PODXL	-1.06
B4DKM6	Serum paraoxonase/arylesterase 2	PON2	-1.06
A0A024RC61	Aminopeptidase N	ANPEP	-1.07
P38117	Electron transfer flavoprotein subunit beta	ETFB	-1.07
A0A096LPI6	ES1 protein homolog, mitochondrial	C21orf33	-1.09
Q6DHZ2	cAMP-dependent protein kinase type II-beta regulatory subunit	PRKAR2B	-1.09
A0A024R9D7	2,4-dienoyl-CoA reductase, mitochondrial	DECR1	-1.09
P49006	MARCKS-related protein	MARCKSL1	-1.10
Q53FJ5	Prosaposin	PSAP	-1.10
B4E324	Carboxypeptidase;	CTSA	-1.10
Q9NV96	Cell cycle control protein 50A	TMEM30A	-1.10
Q14XT3	Cytochrome c oxidase subunit 2	COX2	-1.11
P35580	Myosin-10	MYH10	-1.11
B7Z8A2	Prenylcysteine oxidase 1	PCYOX1	-1.11
Q96CM8	Acyl-CoA synthetase family member 2, mitochondrial	ACSF2	-1.12
Q9Y4D7	Plexin-D1	PLXND1	-1.15
A0A024RAB6	Basement membrane-specific heparan sulfate proteoglycan core protein;Endorepellin;LG3 peptide	HSPG2	-1.16
B2R7D2	Multiple inositol polyphosphate phosphatase 1	MINPP1	-1.16
A0A1B0GW44	Cathepsin D;	HEL-S-130P	-1.17
A0A024RAD8	Delta-1-pyrroline-5-carboxylate dehydrogenase, mitochondrial	ALDH4A1	-1.17
P16284	Platelet endothelial cell adhesion molecule	PECAM1	-1.18
Q9UHL4	Dipeptidyl peptidase 2	DPP7	-1.18
Q8TDB4	Protein MGARP	MGARP	-1.19
P51970	NADH dehydrogenase [ubiquinone] 1 alpha subcomplex subunit 8	NDUFA8	-1.21
Q9BVJ8	Beta-hexosaminidase;Beta-hexosaminidase subunit alpha	HEXA	-1.21
B4DJQ8	Dipeptidyl peptidase 1;	CTSC	-1.23
A8K9J6	CD276 antigen	CD276	-1.23
Q53FB6	Aldehyde dehydrogenase, mitochondrial	ALDH2	-1.25
O75923	Dysferlin	DYSF	-1.25
P04275	von Willebrand factor;von Willebrand antigen 2	VWF	-1.26
P49407	Beta-arrestin-1	ARRB1	-1.27
Q9Y2Q5	Ragulator complex protein LAMTOR2	LAMTOR2	-1.28
Q6IB89	NADH dehydrogenase [ubiquinone] 1 alpha subcomplex subunit 7	NDUFA7	-1.29
Q9NSC5	Homer protein homolog 3	HOMER3	-1.31
Q53H18	Beta-galactosidase	GLB1	-1.32
A0A0C4DFN8	NADPH:adrenodoxin oxidoreductase, mitochondrial	FDXR	-1.34
A8K335	Gamma-glutamyl hydrolase	GGH	-1.34
A0A140VJL0	3-hydroxyisobutyryl-CoA hydrolase, mitochondrial	HIBCH	-1.35
A0A024R8Q1	Lysosomal alpha-glucosidase;	GAA	-1.36
B3KQQ0	Peptidyl-prolyl cis–trans isomerase FKBP9	FKBP9	-1.39
A0A090N7X0	GTPase IMAP family member 4	HIMAP4	-1.39
Q5URX0	Beta-hexosaminidase;	HEXB	-1.41
P36551	Oxygen-dependent coproporphyrinogen-III oxidase, mitochondrial	CPOX	-1.41
A0A024RA75	3-hydroxyisobutyrate dehydrogenase	HIBADH	-1.42
B4DTT0	N-acetylglucosamine-6-sulfatase	DKFZp686E12166	-1.44
A0A090N8H2	GTPase IMAP family member 8	hIAN6	-1.45
Q7Z7M4	Superoxide dismutase;	SOD2	-1.47
Q92626	Peroxidasin homolog	PXDN	-1.48
Q14165	Malectin	MLEC	-1.52
B4DSE2	Tripeptidyl-peptidase 1	TPP1	-1.53
P04040	Catalase	CAT	-1.55
Q96DI8	Heme oxygenase 1	HMOX1	-1.55
B4DJI2	Granulins;	GRN	-1.56
Q5TBU5	Adipogenesis regulatory factor	hCG_1773630	-1.58
P30837	Aldehyde dehydrogenase X, mitochondrial	ALDH1B1	-1.58
Q5NKV8	Intercellular adhesion molecule 1	ICAM1	-1.59
A0A024R5L0	Lysosomal Pro-X carboxypeptidase	PRCP	-1.91
Q8NHP8	Putative phospholipase B-like 2;	PLBD2	-2.01
A0A090N8Z4	GTPase IMAP family member 1	IMAP1	-2.04
K7ERP4	Glutathione peroxidase;	GPX4	-2.18
A0A024RDG9	3-hydroxybutyrate dehydrogenase type 2	BDH2	-2.21
B2R769	Endoplasmic reticulum aminopeptidase 2	ERAP2	-2.53
V9HW74	Ubiquitin carboxyl-terminal hydrolase;	HEL-117	-3.17

Endoglin (ENG, ratio = 3.05), a vascular endothelium glycoprotein was upregulated. It is involved in the positive regulation of angiogenesis, response to hypoxia, positive regulation of collagen biosynthesis and negative regulation of nitric-oxide (NO) synthase processes. Nitric oxide synthase 3 was also highly down expressed (NOS3, ratio = 0.52) indicating low concentration of NO and consequent impairment of vascular remodelling^15,16^ (Table 1).

Von Willebrand Factor (vWF), an important adhesion protein involved in vascular haemostasis, was down regulated (ratio = 0.42), as also validated by RT-PCR analyses (Suppl. Figure 1).

**Figure 2:**
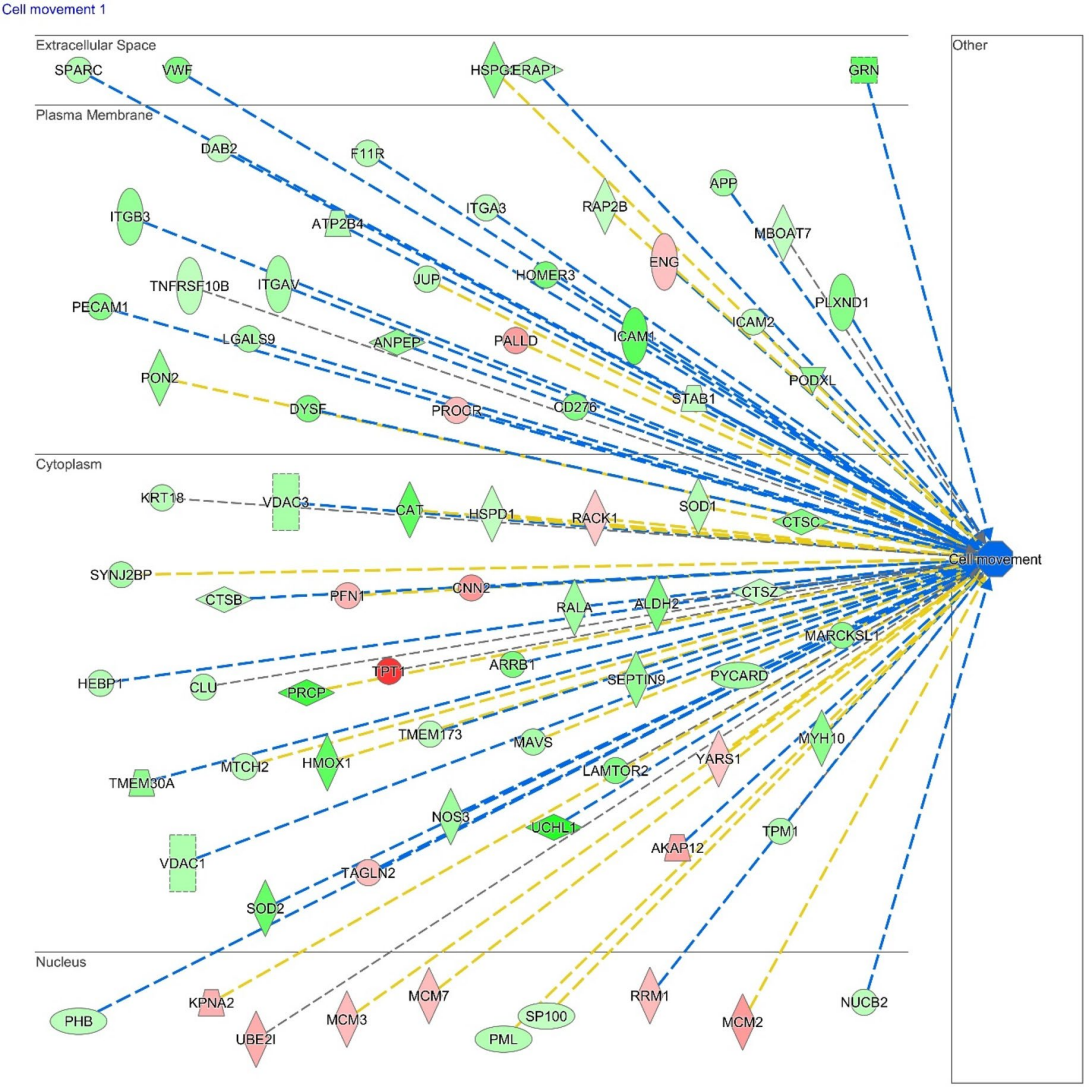
Molecules involved in cell movement significantly altered in CTEPH patients (last version IPA, QIAGEN Inc., https://www.qiagenbioinformatics.com/products/ingenuitypathway-analysis), in red the increased genes, in green those decreased. The color intensity is positive related to the up- or down-gene’s regulation; orange line leads to activation, yellow lines for findings inconsistent with state of downstream molecule; grey line for effect not predicted.

**Figure 3:**
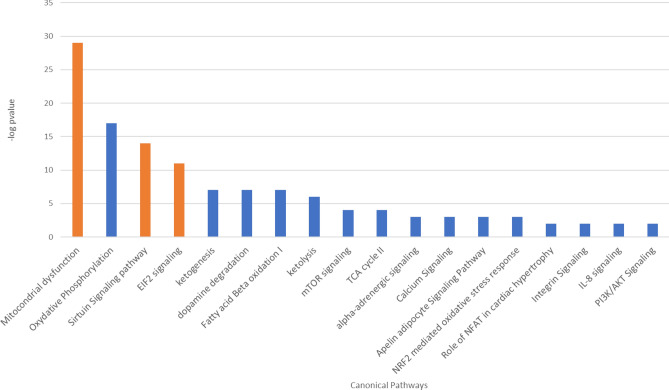
Canonical Pathways that are differentially regulated in CTEPH patients versus controls, in orange the up regulated (z score > 0) and in blue the down regulated ones (zscore < 0) (last version IPA, QIAGEN Inc., https://www.qiagenbioinformatics.com/products/ingenuitypathway-analysis).

Cell movement and angiogenesis functional modules were predicted to be decreased (p value 7.40 e^-05^, 44 genes, z score -2.994), thanks to the contribution of differentially regulated proteins, such as RALA (ratio = 0.52), ERAP2 (ratio = 0.17) TPT1 (ratio = 5.20), MCM2 (ratio = 2.11) and CNN2 (ratio = 2.17) (Fig. 2 and Suppl. Figure 2). The decrease of the calcium signalling pathway (z score –2.23, p-value 2.73 e^-3^, 9 genes) correlates with the decrease of PI3K/AKT, mTOR, NFAT, eiF4 and p70S6K pathways (Fig. 3, Suppl. Table 2). The differentially expressed proteins describing the pathways are directly involved in cell proliferation, essential in the remodelling of vascular endothelium. The regulation of these proteins, such as integrin or phosphatase activator isoforms, disagreed with expression in healthy samples in literature, attesting an overall dysregulation of these pathways in CTEPH disease.

**Figure 4:**
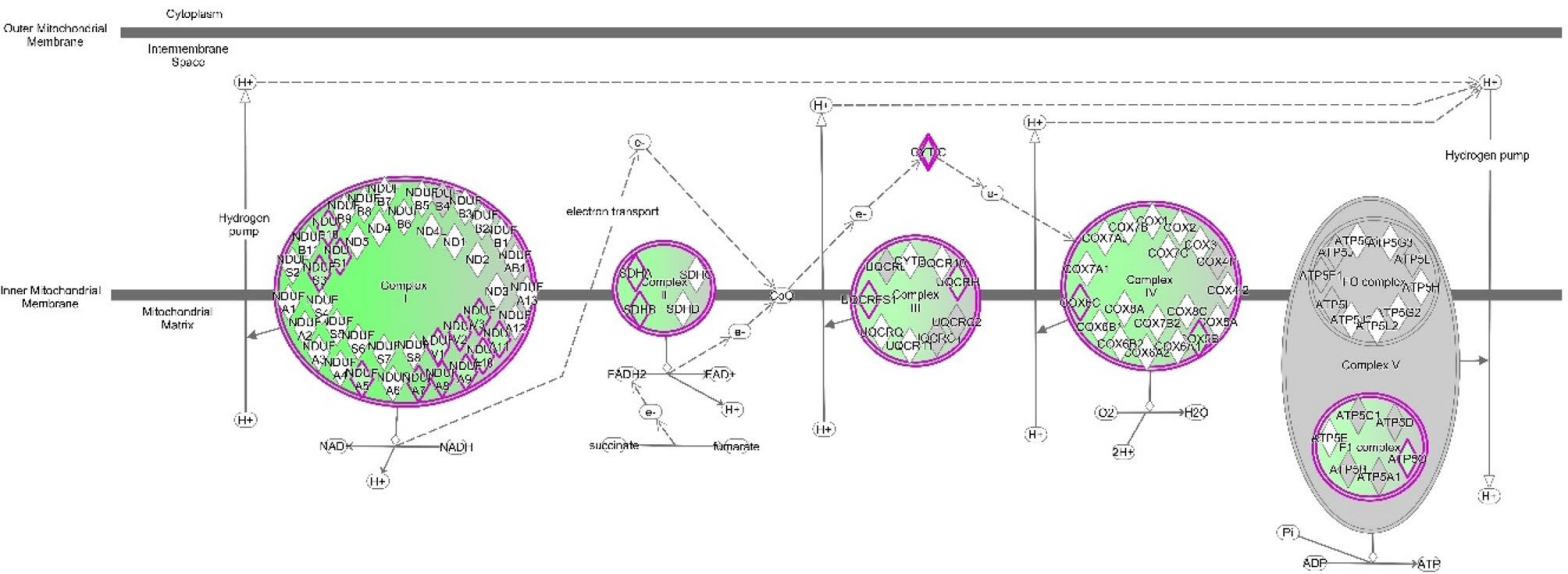
Pathways involved in mitochondrial dysfunction significantly altered in CTEPH patients ((last version IPA, QIAGEN Inc., https://www.qiagenbioinformatics.com/products/ingenuitypathway-analysis), in red the increased genes, in green those decreased. The color intensity is positive related to the up- or down-gene’s regulation; orange line leads to activation, yellow lines for findings inconsistent with state of downstream molecule; grey line for effect not predicted.

**Figure 5:**
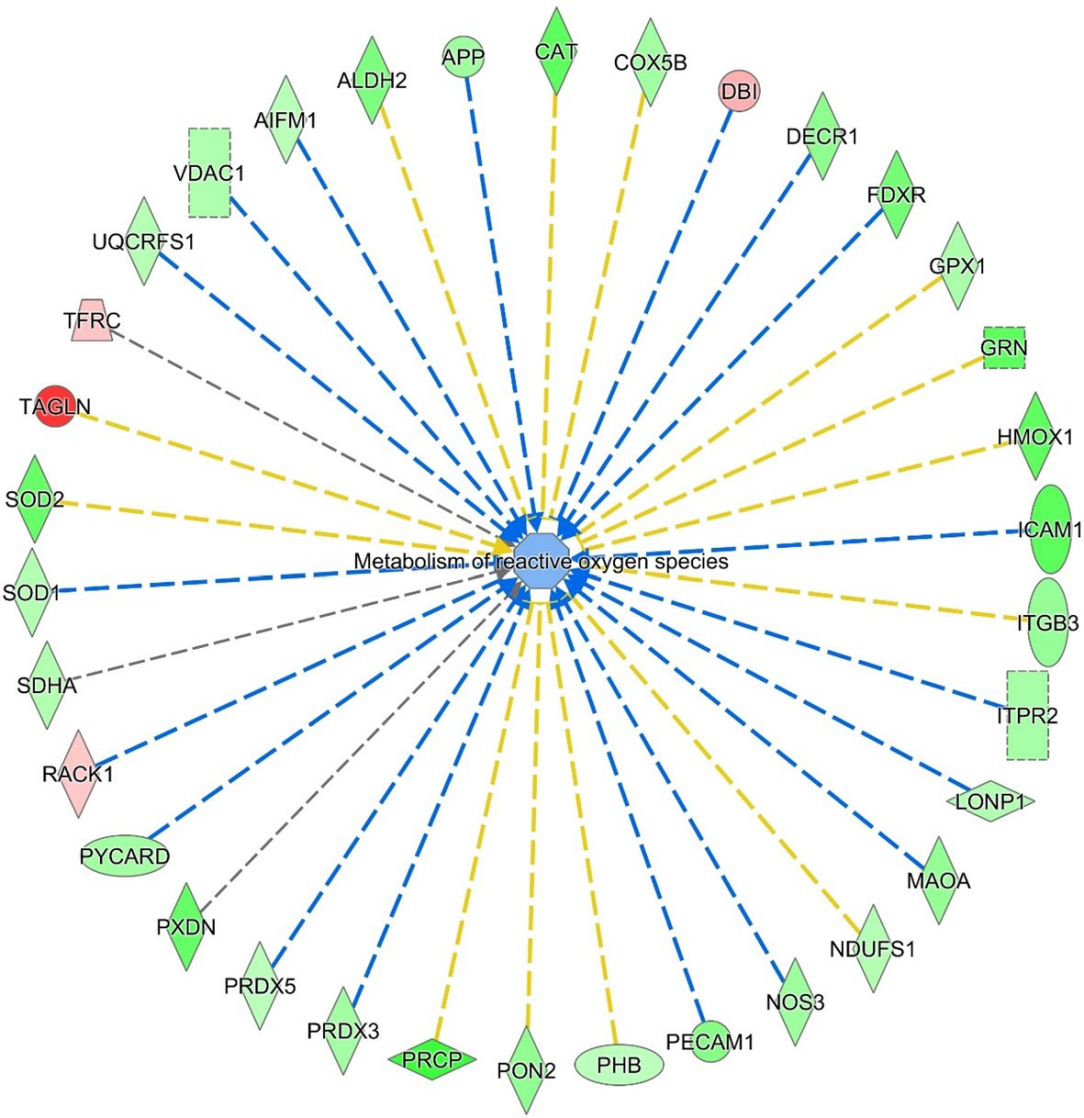
Molecules involved in Metabolism of reactive oxygen species significantly altered in CTEPH patients (IPA), in red the increased genes, in green those decreased.

Protein network analyses revealed a pronounced mitochondrial dysfunction (p-value 3.10 e^-29^, 34 genes). Figure 4 shows the downregulated respiratory and oxidative phosphorylation pathways in mitochondria. Proteins belonging to complexes I, II and III, such as cytochrome c1 (Cyc1, ratio = 0.64), glutathione peroxidase 4 (GPX4, ratio = 0.22), NADH ubiquinone oxidoreductase core subunit A8 (NDUFA8, ratio = 0.43) succinate dehydrogenase complex flavoprotein A (SDHA, ratio = 0.60) were down expressed. GPX4 is an essential antioxidant peroxidase that protect cells from oxidative damage by preventing membrane lipid peroxidation. However, in the present study GPX4 showed a drastic downregulation (ratio = 0.2), suggesting the dysregulation of oxidative stress/damage response. Interestingly the metabolism of reactive oxygen species resulted decreased (z score -0.684, p-value 7.8 e^-10^, 8 genes) (Fig. 5), showing an impaired response to oxidative stress, confirmed by down regulation of NRF2 pathway (z score -1.342, p-value 5.93 e^-3^, 34 genes), principal actor in preventing oxo-damage (Fig. 3, Suppl. Table 2).

mRNA translation was up regulated, as resulted from the increase of EIF2 signaling pathway (p value 1.00 e^-11^, 21 genes, z score 1.941) in which eukaryotic translation initiation factors and ribosomal proteins were over expressed. The down regulation of proteins, such as Lysosomal Pro-X carboxypeptidase (PRCP, ratio = 0.26) induced a decrease of angiogenesis functional module (z score -0.389, p-value 1.72 e^-05^) and cell movement of leukocytes signaling (z score -1.50, p-value 1.12 e^-04^), key components of thrombus resolution.

Fatty acid metabolism (z score -0.933, p-value 2.58 e^-10^, 38 genes) was decreased while the concentration of lipids (z score 1.846, p-value 1.74 e^-6^, 39 genes) was increased, attesting dysfunction in lipid metabolism during CTEPH disease (Suppl. Figure 3 and 4). In addition, inflammation of organ was predicted to be increased (z score 3.147, p-value 5.03 e ^-05^, 24 genes) (Suppl. Figure 5 and Suppl. Table 3).

**Figure 6:**
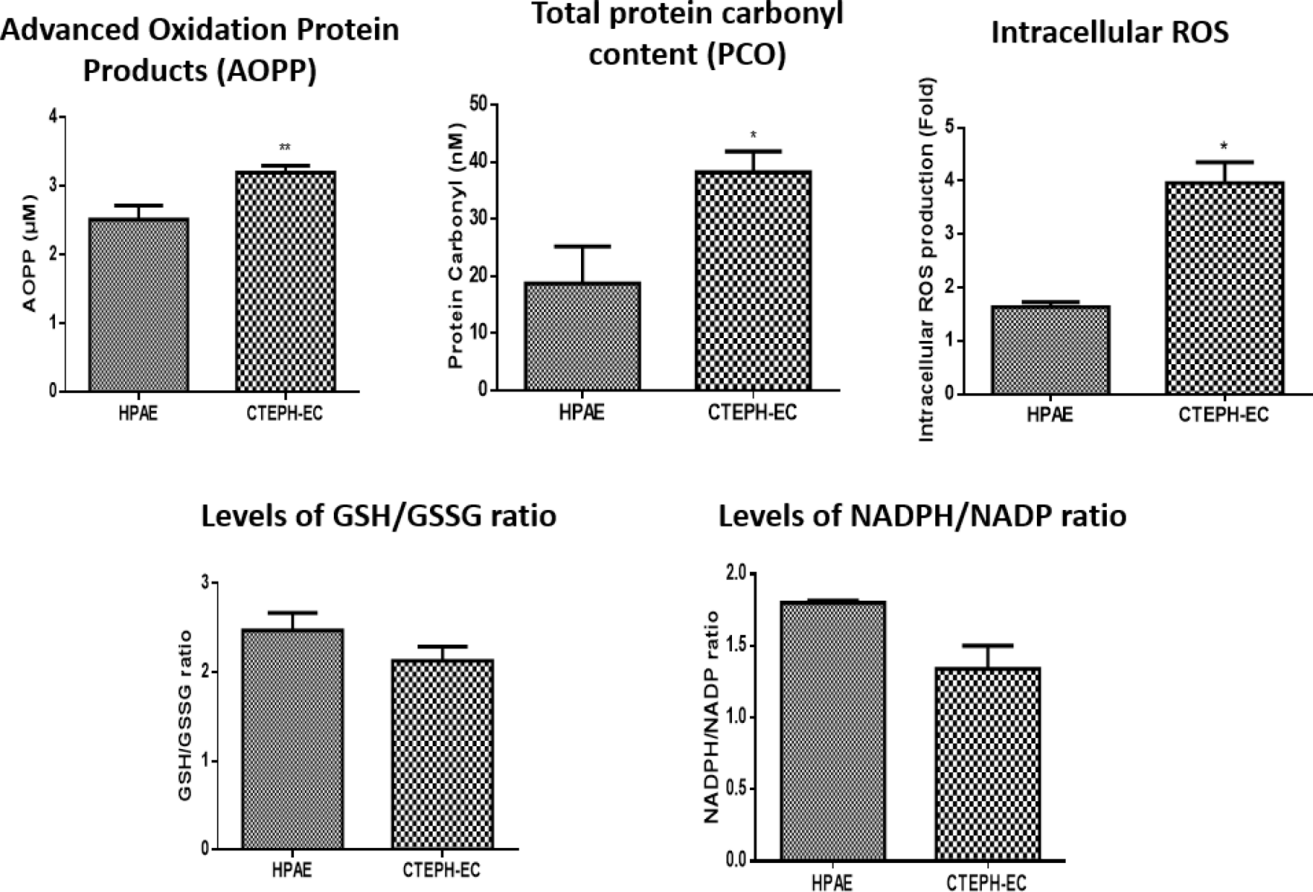
Levels of oxidants and antioxidants in dysfunctional ECs of CTEPH. Data were shown in mean ± SEM. Differences were considered significant when p < 0.05 (*), 0.001 < p < 0.01 (**), p < 0.001 (***), p < 0.0001(****).

To understand the status of oxidants, the levels of advanced oxidation protein products, (AOPPs), total protein carbonyl content (PCO), and intracellular reactive oxygen species (ROS) were quantified. Similarly, to study the status of antioxidant levels the GSH (reduced)/GSSG (oxidized) ratio, and NADPH (reduced)/NADP (oxidized) ratio was measured. As shown in Fig. 6, the levels of AOPPs, total PCOs, and intracellular ROS were increased in dysfunctional ECs of CTEPH, whereas GSH to GSSG and NADPH to NADP ratios were decreased.

## Discussion

CTEPH is a multi-factorial disease and its diagnosis and treatment are complex. This study represents the first functional analyses of protein network changing during CTEPH, using patient-derived cells. The advanced analytical approach in combination with the study of dysregulated pathways allowed the identification of several important abnormal regulated functional modules, such as mitochondrial metabolism, cell proliferation signalling, oxo-response and fatty acid metabolism, proving the presence of endothelial dysfunction.

Akt-mTOR pathway has multiple downstream effects, related to different cell and tissue types. In CTEPH and related diseases this pathway is activated by thrombin, inducing an enhance of calcium concentration, cell proliferation and endothelium remodelling^16^. In this study the differentially regulated proteins, such as integrin alpha, nitric oxide synthase and protein phosphatase 2, inhibited cell proliferation, showed by the dysregulation of AKT-mTOR, calcium, integrin and NFAT network modules. To date, mTOR has gained wide interest in its potential therapeutic role in several proliferative diseases such as cancer. The role of mTOR in pulmonary hypertension it is not fully understood. Previous work showed an obvious downregulation of of mTOR in lung tissues from patients with pulmonary arterial hypertension (PAH) which was reverted in specific adenovector, mTOR overexpressing mice^17^. After mTOR rebalance mice showed a marked reduction in right ventricular hypertrophy and a reduction of pulmonary artery remodelling. It is not known whether the same phenomena could be happening in CTEPH patients. Another work showed that smooth muscle cells and/or myofibroblasts isolated from pulmonary endarterectomy specimens presented higher expression levels of PDGF and PDGF receptors known to potently activate the Akt/mTOR pathway^18^. CTEPH cells increased phosphorylation of Akt, which subsequently caused an increase in phosphorylated mTOR and p70S6K and that could be inhibited by rapamycin. The levels of mTOR compared to healthy pulmonary artery smooth muscle cells or endothelial cells without PDGF stimulation are not clear to confirm the basal levels of mTOR in patients with CTEPH vs control cells. Additionally, Ogawa et al. work significantly differs with the present findings as the cell population isolated from the pulmonary endarterectomy specimens is different^18^. In the present study only endothelial cells and not smooth muscle cells or myofibroblasts were isolated. Despite both cell lines co-exist in pulmonary arteries and interact constantly between each other both cells play different roles in vascular homeostasis. This could explain why the expression levels of mTOR in endothelial CTEPH cells are different to smooth muscle cells. However, this protein functional module can be targeted by drug or an indication for early diagnosis of CTEPH disease since its activation allows the remodelling of vascular endothelium.

The quantitative proteomics approach showed for the first time the protein PLOD2 as the most upregulated protein attesting its key role in CTEPH. PLOD2 is an attachment site for carbohydrates and it is involved in the stability of collagen fibrils. Collagen plays a key role in the maintenance of integrity and elasticity of normal vessel walls as well as atherosclerotic vessel walls and it stimulates the thrombosis formation and scar formation processes particularly in PAH patients^19^. Under the hypoxic conditions, PLOD2 mediate the remodelling of extracellular matrix in synergy with collagen prolyl hydroxylases (P4HA1 and P4HA2)^20^. PLOD2 is involved in collagen deposition that induces the reduction of vascular elasticity^20^. PLOD2 promotes the cross-link of collagen, important event for the formation and stabilization of insoluble collagen fibrils. Collagen containing pyridinoline cross-links are difficult to degrade by collagenases, increasing collagen deposition and tissue stiffness. Over-expression of PLOD2 and over-production of insoluble collagen fibres in CTEPH disease could increase the collagen ‘s deposition, tissue’s stiffness, myofibroblast’s differentiation and platelet’s aggregation^13,14^. COL18A1, involved in the maintenance of vessel wall integrity and structure in atherogenesis^20,21^, was down regulated. On the other hand, TAGLN showed an extensive upregulation with a ratio of 5.3 in CTEPH-ECs. Recent study has shown that TAGLN overexpression promoted the proliferation and migration of human pulmonary arterial smooth muscle cells (PASMC), strengthened cytoskeleton; suppression of this protein activated PASMC apoptosis, reducing cell proliferation and migration^22^. The higher expression of TAGLN might be related to the hyperproliferative phenotype of CTEPH-ECs and it might have an important role in endothelial dysfunction. Furthermore, a drastic alteration in mitochondrial respiratory chain complexes was described by network analyses.

Haemolysis and oxidative stress are peculiar features in pulmonary hypertension and endothelial dysfunction^23–27^. Glutathione, a major non-protein thiol in living organisms, plays a central role in coordinating the body's antioxidant defence processes^28–30^. Perturbation of glutathione status of a biological system has been reported to lead to serious consequences. In the present study, low level of GSH in CTEPH dysfunctional ECs was observed. Interestingly quantitative proteomics analysis showed a down-regulation of GPX4 and GPX1 proteins that catalyse the formation of glutathione disulphide in presence of hydrogen peroxide (Table 1). GPX1 decreased the bioavailability of NO and increased the oxidative stress^31^. Therefore, the underlying mechanisms might be attributed to the unavailability of GSH, the substrate for GPX. The capacity of the glutathione system to deal with free radicals mainly depends upon the activity of peroxidase and glutathione reductase (GR). Glutathione reductase keeps a high ratio of GSH/GSSG in normal cells. In CTEPH-ECs glutathione reductase might be in lower levels, which in turn is linked to the decreased concentration of GPx and reduced glutathione. The down regulation of identified GPX4, GPX1, NOS3 proteins attested the increment of oxidative stress. Among these GPX4 found as one of the most down-regulated protein. Under physiological conditions, the antioxidants related to glutathione peroxidase family proteins GPX1 and GPX4 protects the cells from oxidative stress^32^. GPX4 prevents the cells from ferroptosis, a non-apoptotic cell death as consequence of iron-dependent accumulation of lipid reactive oxygen species (ROS)^33^. Elevated levels of GPX1 protected the cultured HPAECs from hydrogen peroxide induced cell death^34^. Reduced expression of GPX1 can also lead to inflammatory activation of ECs, that further increase to a proatherogenic endothelial phenotype^35^. Genetic deletion of GPX1 decreased bioavailability of NO and increased the oxidative stress^35^. Moreover, normal ECs always generate endothelial nitric oxide synthase (eNOS), which is essential to maintain various endothelial functions including the vascular smooth muscle relaxation, vascular endothelial growth factor (VEGF) induced angiogenesis, suppression of inflammatory cell migration and adhesion, angiogenesis, inhibition of thrombosis and coagulation. The impairment of eNOS causes the endothelial dysfunction and oxidative stress which further increases the atherogenesis. The endothelial NOS3 protein, responsible to produce NO, was found down regulated and vascular endothelium glycoprotein ENG, involved in the negative regulation of NO production, was found up regulated. Therefore, the reduction in NO bioavailability, resulting from reduced NO production and/or increased NO degradation by superoxide anion, describes the onset of endothelial dysfunction^27^.

Moreover, NADPH oxidase-derived ROS play a role in vascular pathology as well as in the maintenance of normal physiological vascular function^36^. NADPH has been suggested to act as an indirectly operating antioxidant, thus maintaining the antioxidative power of glutathione^37^. In the present study, lower level of NADPH was observed in CTEPH, suggesting the increasing of oxidative stress in dysfunctional ECs.

The increased levels of AOPPs might explain the involvement of higher global inflammatory activity in endothelial dysfunction^38^. Increased AOPPs production might be involved in the generation of various types of ROS^39^. The accumulation of ROS has been well established in the pathogenesis of several diseases including diabetes^40^, atherosclerosis^41^, renal ischemia^42^, inflammatory diseases^43^ and cancer^44^. Increased production of ROS in dysfunctional ECs might enhance pro-inflammatory pathways and affect protein, lipid and enzymatic activity.

In summary, the mechanisms underlying endothelial dysfunction implicates the alterations in metabolism, inflammation and oxidative stress events.

## Conclusions

This is the first study to attempt the description of molecular network in in endothelial dysfunction associated with CTEPH using patient-derived pathological ECs. Quantitative proteomic profiling in endothelial dysfunction reveals the differentially regulated proteins that might behave as a biomarker signature of vascular injury, metabolism and oxidative stress. Increased production of oxidants and decreased production of antioxidant biomarkers as well as down regulation of protein which had an antioxidant property strongly explained the correlation of the role of oxidative stress in endothelial dysfunction of CTEPH.

In conclusion the study unveiled the pathophysiological mechanisms and some important functional modules, constituted by differentially regulated proteins, dysregulated during the disease.

### Potential limitations and future perspective of the study

Although we used a statistically significant number of patients-derived ECs to identify underlying mechanisms associated with endothelial dysfunction of CTEPH, further experiments need to be performed to validate the expression of proteins using clinical samples e.g. blood, for determining them as a potential prognostic marker for early prediction of endothelial dysfunction. Despite knowing the background of patients from whom we have collected specimens to isolate ECs, the long-term medical history and potential risk factors to which they might have exposed is anonymous. The perspective of the current study would be to perform more experiments to unveil mechanistic and functional characteristics of PLOD2 and ENG1, which might provide an in-depth understanding of endothelial dysfunction. To understand the effect of these dysregulated proteins in downstream signaling pathways, gene overexpression and knockdown analysis will be performed in vitro. Additionally, to understand whether the upregulation of PLOD2 and ENG1 might play a key role in inducing oxidative stress, we will use various biochemical and molecular biology approaches followed by gene overexpression in ECs. To further overcome the limitations of the current study, perspective studies would be performed using patient-specific induced pluripotent stem cell-derived endothelial cells (iPSC-ECs).

## Materials and methods

### Endothelial cell culture

Commercially available human pulmonary artery endothelial cells (HPAE) were used as controls. Five individual ECs lines, derived from surgical specimens obtained in patients with CTEPH (CTEPH-ECs) who underwent pulmonary endarterectomy, were received from Dr. Olga Tura, Research Scientist, Department of Pulmonary Medicine, Hospital Clínic-Institut d'Investigacions Biomèdiques August Pi iSunyer (IDIBAPS), University of Barcelona, Spain. The study was conducted in accordance with the Declaration of Helsinki, was approved by the Committee on Human Research of our institution and all subjects gave written informed consent (Comité Ético de Investigación Clínica del Hospital Clínic de Barcelona). The clinical characteristics of CTEPH endothelial cells are summarized in Supplementary Table 4.

The specimens containing thromboembolic material with clear thrombus were obtained after pulmonary endarterectomy (PEA) surgery from CTEPH patients at IDIBAPS. Material were washed with PBS and minced into 1–2 mm pieces. Explants were seeded into 0.2% gelatin-coated 6-well plates and maintained under standard cell culture conditions. After 24 h, tissue explants, non-adherent cells, and debris were aspirated. Medium was changed every other day until the first passage of pulmonary endothelial outgrowth cells emerged and cells were cultured as previously described^45^.

### Protein extraction and In-solution trypsin digestion

Confluent cells were trypsinized and collected by centrifugation at 1200 rpm for 5 min. Harvested cells were washed two times with cold PBS. The cell pellets were resuspended in the solubilization buffer (8 M urea in 50 mM Tris–HCl, 30 mM NaCl, pH 8.5 and 1% protease inhibitor) and incubated on ice for 5–10 min. Cell lysates were further homogenized by sonication in an ice bath for three times each for 5 s with 30 s intervals, using an ultra sonicator. Samples were centrifuged at 14,000 rpm for 20 min at 4ºC. The protein supernatant was collected into the new Eppendorf tube and pelleted cell debris was discarded. Samples were stored at -80 ºC until we use it for further experiments. The protein estimation was carried out by using the Bradford assay.

10 µg of total protein was resuspended in 50 mM ammonium bicarbonate. Reduction was carried out by incubating 5 mM final concentration of DTT for 30 min at 52 °C, followed by an alkylation with 15 mM final concentration of iodoacetamide for 20 min in the dark at room temperature. Trypsin digestion was allowed at 37 °C overnight, with an enzyme: substrate ratio of 1:10. The resulting peptides were used for MS analysis.

### Mass spectrometry and data analyses

Tryptic peptides were analyzed using a Dionex Ultimate 3000 nano-LC system (Sunnyvale CA, USA) connected to an Orbitrap Fusion Tribrid Mass Spectrometer (Thermo Scientific, Bremen, Germany) equipped with a nano-electrospray ion source. Peptide mixtures were pre-concentrated onto an Acclaim PepMap 100 – 100 µm × 2 cm C18 and separated on EASY-Spray column, 15 cm × 75 µm ID packed with Thermo Scientific Acclaim PepMap RSLC C18, 3 µm, 100 Å. The temperature was setting to 35 °C and the flow rate was 300 nL/min. Mobile phases were the following: 0.1% Formic acid (FA) in water (solvent A); 0.1% FA in water/acetonitrile (solvent B) with 2/8 ratio. Peptides were eluted from the column with the following gradient: 4% to 28% of B for 90 min and then 28% to 40% of B in 10 min, and to 95% within the following 6 min to rinse the column. Column was re-equilibrated for 20 min. Total run time was 130 min. One blank was run between triplicates to prevent sample carryover. MS spectra were collected over an m/z range of 375–1500 at 120,000 resolutions, operating in the data dependent mode, cycle time of 3 s. Higher-energy collisional dissociation (HCD) was performed with collision energy set at 35%. Each sample was analysed in three technical triplicates^45^.

Resulting MS raw files from all the technical and biological replicates were analysed by using MaxQuant software^48^ (version 1.6.2.3). Andromeda search engine was used to identify MS/MS based peptide and proteins in MaxQuant comprises a target-decoy approach with less than 1% of False Discovery Rate (FDR). In the present study we used *Uniprot_Homosapiens* database. Trypsin was used for enzyme specificity, 2 missed cleavages and maximum five number of modifications per peptide was allowed. Methionine oxidation and acetylation (N terminus) was used as a variable modification. Carbamidomethylation was used as a fixed modification. The proteins were selected with a minimum of two peptides. For the label-free quantification of proteins, we applied MaxLFQ algorithm. Match between the runs option was enabled and remaining default parameters were permitted. Data available on request from the authors.

An open source Perseus software^46^ (version 1.6.1.3; Max Planck Institute of Biochemistry, Germany) was used for the identification of statistically significantly differentially regulated proteins. The interpretation and visualization of results obtained from MaxQuant software were performed by a two-sample t-test using Perseus (v1.6.1.3, Max Planck Institute of Biochemistry, Germany). Statistical parameters (p < 0.05; q < 0.05, q = FDR adjusted p-value) were set to identify the differentially expressed proteins between samples, categorized in two groups: CTEPH-EC (patients) and HPAEC (controls) (log2 ratio). Variabilities of biological replicates were measured with Pearson correlation coefficient values of the LFQ intensities. The differentially regulated proteins with a minimum of two peptides and FDR adjusted p-value < 0.05 were considered as statistically significant^46^.

### Protein network analysis

The network protein analysis related to significantly altered proteins was carried out by IngenuityPathwaysAnalysis(IPA)(QIAGENInc., https://www.qiagenbioinformatics.com/products/ingenuitypathway-analysis). The statistical enrichment of involved pathways is performed by the right-tailed Fisher’s exact test, in correlation with QIAGEN Knowledge Base, assigning a p-value. The core analyses performed by IPA, using the differentially expressed proteins in the uploaded dataset, assess signaling pathways, molecular interaction network and biological functions that are likely to be perturbed. The overall activation/inhibition states of canonical pathways are predicted based on a z-score algorithm. This z-score is used to statistically compare the uploaded dataset with the pathway patterns. The pathways are colored to indicate their activation z-scores: orange predict a gain of function, while blue a loss of function. The pathway is activated when molecules’ causal relationships with each other (i.e., activation edge and the inhibition edge between the molecules based on literature findings) generate an activity pattern for the molecules and the end-point functions in the pathway.

### Quantitative gene expression analysis

Total RNA extraction and conversion to first-strand cDNA were done using iScript RT-qPCR sample preparation reagent (Bio-Rad) and Maxima First Strand cDNA Synthesis Kit (Thermo Scientific), respectively according to the manufacturer’s protocol. Quantitative RT-PCR analysis was performed using an ABI PRISM 7000 Sequence detection system cDNA was amplified in triplicate using Maxima SYBR Green/ROX qPCR Master Mix (Thermo Scientific). The thermal cycling programme started at 95 °C for 10 min for enzyme activation, followed by 40 cycles of amplification at 95 °C for 15 s, followed by 60 °C for 1 min. Gene expression was normalized to the housekeeping gene RPLP0. Relative expression of targeted genes was determined using the 2^-ΔΔCT^ method. P value below 0.05 were considered as statistically significant.

### Biomolecular assays

Advanced oxidation protein product (AOPP) levels were analysed by using the OxiSelect AOPP kit (STA318, Cell Biolabs, San Diego, CA, USA) following the manufacturer’s protocol. Briefly, the confluent cells were collected, lysed in buffer (150 mM NaCl, 1% NP-40, 0.5% sodium deoxycholate, 0.1% SDS, 50 mM Tris, pH 8, 5 mM EDTA and protease inhibitors) and centrifuged for 20 min at 14,000 rpm. The pellet containing cell debris was discarded; the protein supernatant was collected and quantified by Bradford assay. 50 µg of protein in 200 µL final volume of control and pathological cell lysates were subjected to10 µL of chloramine reaction initiator followed by 20 µL of stop solution. Absorbance was recorded at 340 nm by using the spectrophotometric plate reader (PowerWave biotek). Cell lysates used in this assay was prepared from the ECs in passages between 6–9. Experiments were performed with biological and technical replicates^47^.

Protein carbonyl content, a general indicator of protein oxidation was measured fluorometrically (480 nm excitation/530 nm emission) using a commercial kit (Cell Biolabs, OxiSelect No. STA-307) as per the instructions provided by the manufacturer. Experiments were performed with biological and technical replicates.

ROS was measured by OxiSelect ROS Assay Kit (Cell Biolabs, STA-342) as per the manufacturer protocol. Briefly, the control and pathological ECs were grown in 96 well plate at a density of 9 × 10^3^ cells and incubated with the dichloro-dihydro-fluorescein diacetate (DCFH-DA) containing medium at 37 °C for 60 min in the dark. The confluent cells were lysed, and the fluorescence was recorded by using the fluorometric plate reader at 480 nm/530 nm. Experiments were performed with biological and technical replicates.

GSH/GSSG ratio in control and pathological EC cultures was determined using GSH/GSSG Ratio Detection Assay Kit (Fluorometric-Green) (Abcam Inc. #ab138881) according to the manufacturer’s guidelines. The samples were prepared by lysis of total cell protein in 0.5% NP-40 lysis buffer followed by a dilution of 1:50 for GSH analysis. In brief, serial dilution of GSH and GSSG stock standards were prepared along with assay mixtures for detection of GSH and total GSH using 100 × Thiol green stock solutions, assay buffer and GSSG probe. A one- step fluorometric reaction of sample with respective assay buffers were incubated for 30 min. Fluorescence intensity was then monitored at Ex/Em of 490/520 nm. GSSG was determined by subtracting GSH from total GSH. Finally, the ratio of GSH was plotted against GSSG to obtain the GSH activity^48^. Experiments were performed with biological and technical replicates.

NADPH/NADP ratio in control and pathological ECs was determined by using NADP/NADPH quantification kit (Sigma) as per the manufacturer’s protocols. Briefly, 1 × 10^6^ confluent cells were pelleted and washed with cold PBS. Cells were extracted with 800 µl of NADP/NADPH extraction buffer and allowed to incubation for 10 min on ice. Samples were centrifuged at 10,000*g* for 10 min and supernatant used for determining the ratio of NADP and NADPH. To remove the enzymes that may consume NADPH rapidly were removed by filtering through a 10kDA cut-off spin filter (Millipore, UFC501096). An aliquot of the supernatant was heated at 60 °C for 30 min to decompose the NADP^+^, cooled on ice, and spun quickly to remove the precipitate. Another aliquot of the supernatant was not heated. Both aliquots were reacted with NADP^+^ cycling buffer and enzyme mix (containing glucose-6-phosphate dehydrogenase (G6PDH) for 5 min at room temperature to convert NADP^+^ to NADPH. The solutions were then incubated with NADPH developer for 2 h and the absorbance measured at 450 nm. The amount of NADPH (heated sample) and the total NADP^+^ and NADPH (unheated sample) were quantified from an NADPH standard curve^49^. Experiments were performed with biological and technical replicates.

## Supplementary Information

Supplementary Table 1.

Supplementary Table 2.

Supplementary Table 3.

Supplementary Table 4.

Supplementary Figures.

## References

[1] Kim NH (2019). Chronic thromboembolic pulmonary hypertension. Eur. Respir. J..

[2] Wynants M (2012). Effects of C-reactive protein on human pulmonary vascular cells in chronic thromboembolic pulmonary hypertension. Eur. Respir. J..

[3] Bonderman D (2008). Role for staphylococci in misguided thrombus resolution of chronic thromboembolic pulmonary hypertension. Arterioscler. Thromb. Vasc. Biol..

[4] Albani S, Biondi F, Stolfo D, Lo Giudice F, Sinagra G (2019). Chronic Thromboembolic Pulmonary Hypertension (CTEPH). J. Cardiovasc. Med..

[5] Dorfmüller P (2014). Microvascular disease in chronic thromboembolic pulmonary hypertension: a role for pulmonary veins and systemic vasculature. Eur. Respir. J..

[6] Lum H, Roebuck KA (2001). Oxidant stress and endothelial cell dysfunction. Am. J. Physiol. Physiol..

[7] Ranchoux, B., et al. Endothelial dysfunction in pulmonary arterial hypertension: an evolving landscape (2017 Grover Conference Series). *Pulmonary Circulation*. SAGE Publications Ltd January 1 (2018). 10.1177/2045893217752912 PMC5798691 29283043

[8] Makino A, Firth AL, Yuan JXJ (2011). Endothelial and smooth muscle cell ion channels in pulmonary vasoconstriction and vascular remodeling. Compr. Physiol..

[9] Hoeper MM (2014). Chronic thromboembolic pulmonary hypertension. Lancet. Respir. Med..

[10] Kim NH (2013). Chronic thromboembolic pulmonary hypertension. J. Am. Coll. Cardiol..

[11] Konstantinides S (2014). 2014 ESC guidelines on the diagnosis and management of acute pulmonary embolism. Kardiol. Pol..

[12] Zhang Y (2019). Advances in targeted therapy for chronic thromboembolic pulmonary hypertension. Heart Fail. Rev..

[13] Mia MM, Bank RA (2016). The pro-fibrotic properties of transforming growth factor on human fibroblasts are counteracted by caffeic acid by inhibiting myofibroblast formation and collagen synthesis. Cell Tissue Res..

[14] Van Der Slot AJ (2005). Elevated formation of pyridinoline cross-links by profibrotic cytokines is associated with enhanced lysyl hydroxylase 2b levels. Biochim. Biophys. Acta Mol. Basis Dis..

[15] Ogawa A (2013). Thrombin-mediated activation of Akt signaling contributes to pulmonary vascular remodeling in pulmonary hypertension. Physiol. Rep..

[16] Simonneau G (2017). The pathophysiology of chronic thromboembolic pulmonary hypertension. Eur. Respir. Rev..

[17] Li L (2015). Mammalian target of rapamycin overexpression antagonizes chronic hypoxia-triggered pulmonary arterial hypertension via the autophagic pathway. Int. J. Mol. Med..

[18] Ogawa A (2009). Inhibition of mTOR attenuates store-operated Ca2+ entry in cells from endarterectomized tissues of patients with chronic thromboembolic pulmonary hypertension. Am. J. Physiol. Lung. Cell. Mol. Physiol..

[19] Zhao YD (2015). A biochemical approach to understand the pathogenesis of advanced pulmonary arterial hypertension: metabolomic profiles of arginine, sphingosine-1-phosphate, and heme of human lung. PLoS ONE.

[20] Gilkes DM, Bajpai S, Chaturvedi P, Wirtz D, Semenza GL (2013). Hypoxia-inducible factor 1 (HIF-1) promotes extracellular matrix remodeling under hypoxic conditions by inducing P4HA1, P4HA2, and PLOD2 expression in fibroblasts. J. Biol. Chem..

[21] Xu R (2001). NC1 domain of human type VIII collagen (Alpha 1) inhibits bovine aortic endothelial cell proliferation and causes cell apoptosis. Biochem. Biophys. Res. Commun..

[22] Huang L (2018). Transgelin as a potential target in the reversibility of pulmonary arterial hypertension secondary to congenital heart disease. J. Cell. Mol. Med..

[23] Machado RF, Gladwin MT (2010). pulmonary hypertension in hemolytic disorders: pulmonary vascular disease: the global perspective. Chest.

[24] Minneci PC (2005). Hemolysis-associated endothelial dysfunction mediated by accelerated NO inactivation by decompartmentalized oxyhemoglobin. J. Clin. Invest..

[25] Mathew R, Huang J, Wu JM, Fallon JT, Gewitz MH (2016). Hematological disorders and pulmonary hypertension. World J. Cardiol..

[26] Cai H, Harrison DG (2000). Endothelial dysfunction in cardiovascular diseases: the role of oxidant stress. Circ. Res..

[27] Incalza MA (2018). Oxidative stress and reactive oxygen species in endothelial dysfunction associated with cardiovascular and metabolic diseases. Vascul. Pharmacol..

[28] Kidd PM (1997). Glutathione: systemic protectant against oxidative and free radical damage. Altern Med Rev..

[29] McLeay Y, Stannard S, Houltham S, Starck C (2017). Dietary thiols in exercise: oxidative stress defence, exercise performance, and adaptation. J. Int. Soc. Sports Nutr..

[30] Tardiolo G (2018). Overview on the effects of N-acetylcysteine in neurodegenerative diseases. Molecules.

[31] Higashi Y, Pandey A, Goodwin B, Delafontaine P (2013). Insulin-like growth factor-1 regulates glutathione peroxidase expression and activity in vascular endothelial cells: implications for atheroprotective actions of insulin-like growth factor-1. Biochim. Biophys. Acta.

[32] Yang WS (2014). Regulation of ferroptotic cancer cell death by GPX4. Cell.

[33] Zhang Y, Handy DE, Loscalzo J (2005). Adenosine-dependent induction of glutathione peroxidase 1 in human primary endothelial cells and protection against oxidative stress. Circ. Res..

[34] Barroso M (2014). Inhibition of cellular methyltransferases promotes endothelial cell activation by suppressing glutathione peroxidase 1 protein expression. J. Biol. Chem..

[35] Forgione MA (2002). Cellular glutathione peroxidase deficiency and endothelial dysfunction. Am. J. Physiol. Circ. Physiol..

[36] Konior A, Schramm A, Czesnikiewicz-Guzik M, Guzik TJ (2014). NADPH Oxidases in vascular pathology. Antioxid. Redox Signal..

[37] Kirsch M, De Groot H (2001). NAD(P)H, a directly operating antioxidant?. FASEB J..

[38] Skvarilová M, Bulava A, Stejskal D, Adamovská S, Bartek J (2005). Increased level of advanced oxidation products (AOPP) as a marker of oxidative stress in patients with acute coronary syndrome. Biomed. Pap. Med. Fac. Univ. Palacky. Olomouc. Czech. Repub..

[39] Vona R (2018). Oxidative stress in the pathogenesis of systemic scleroderma: an overview. J. Cell. Mol. Med..

[40] Fakhruddin S, Alanazi W, Jackson KE (2017). Diabetes-induced reactive oxygen species: mechanism of their generation and role in renal injury. J. Diabetes Res..

[41] Kattoor AJ, Pothineni NVK, Palagiri D, Mehta JL (2017). Oxidative stress in atherosclerosis. Curr. Atheroscler. Rep..

[42] Granger DN, Kvietys PR (2015). Reperfusion injury and reactive oxygen species: the evolution of a concept. Redox Biol..

[43] Forrester SJ, Kikuchi DS, Hernandes MS, Xu Q, Griendling KK (2018). Reactive oxygen species in metabolic and inflammatory signaling. Circ. Res..

[44] Kumari S, Badana AK, Malla R (2018). Reactive oxygen species: a key constituent in cancer survival. Biomark. Insights.

[45] Nukala SB (2019). Differentially expressed proteins in primary endothelial cells derived from patients with acute myocardial infarction. Hypertension.

[46] Cox J, Mann M (2008). MaxQuant enables high peptide identification rates, individualized p.p.b.-range mass accuracies and proteome-wide protein quantification. Nat. Biotechnol..

[47] Thurmond P, Yang JH, Li Y, Lerner LB, Azadzoi KM (2015). Structural modifications of the prostate in hypoxia, oxidative stress, and chronic ischemia. Korean J. Urol..

[48] Naik P, Sajja RK, Prasad S, Cucullo L (2015). Effect of full flavor and denicotinized cigarettes exposure on the brain microvascular endothelium: a microarray-based gene expression study using a human immortalized BBB endothelial cell line. BMC Neurosci..

[49] Lyu Z (2018). PPARγ maintains the metabolic heterogeneity and homeostasis of renal tubules. EBioMedicine..

