# Supplementary material for: Protein network analyses of pulmonary endothelial cells in chronic thromboembolic pulmonary hypertension
Source: Sci Rep. 2021 Mar 10;11:5583. doi: 10.1038/s41598-021-85004-z (PMC7946953; doi:10.1038/s41598-021-85004-z)
Supplement: Supplementary file 4 — Supplementary Table 4. [file 41598_2021_85004_MOESM4_ESM.doc]

**Supplementary Table 4. Clinical characteristics of CTEPH**

Sarath Babu Nukala, Olga Tura-Ceide, Giancarlo Aldini, Valérie Smolders, Isabel Blanco, Victor I. Peinado, Manuel Castellà, Joan Albert Barberà, Alessandra Altomare, Giovanna Baron, Marina Carini, Marta Cascante and Alfonsina D’Amato.

| **Variables** | **CTEPH** |
| --- | --- |
| Age, years | 59 ± 7 |
| Male sex, n (%) | 4 (80%) |
| Body mass index, Kg/m^2^ | 28.8 ± 2.7 |
|  |  |
|  |  |
|  |  |
|  |  |
| 6MWD, m | 438 ± 98 |
| BNP, pg/mL | 67 ± 99 |
|  |  |
| mPAP, mmHg | 68 ± 8 |
| CI, L/min/m^2^ | 2.37 ± 0.42 |
| PVR, WU | 6.5 ± 3.2 |
| PAWP, mmHg | 8 ± 4 |
|  |  |

Definition of abbreviations: Chronic thromboembolic pulmonary hypertension (CTEPH); brain natriuretic peptide (BNP), mean pulmonary artery pressure (mPAP), cardiac index (CI), pulmonary vascular resistance (PVR), pulmonary artery wedge pressure (PAWP). Values expressed as mean±SD
