# Supplementary material for: Protein network analyses of pulmonary endothelial cells in chronic thromboembolic pulmonary hypertension
Source: Sci Rep. 2021 Mar 10;11:5583. doi: 10.1038/s41598-021-85004-z (PMC7946953; doi:10.1038/s41598-021-85004-z)
Supplement: Supplementary file 5 — Supplementary Figures. [file 41598_2021_85004_MOESM5_ESM.pptx]

## Slide 1
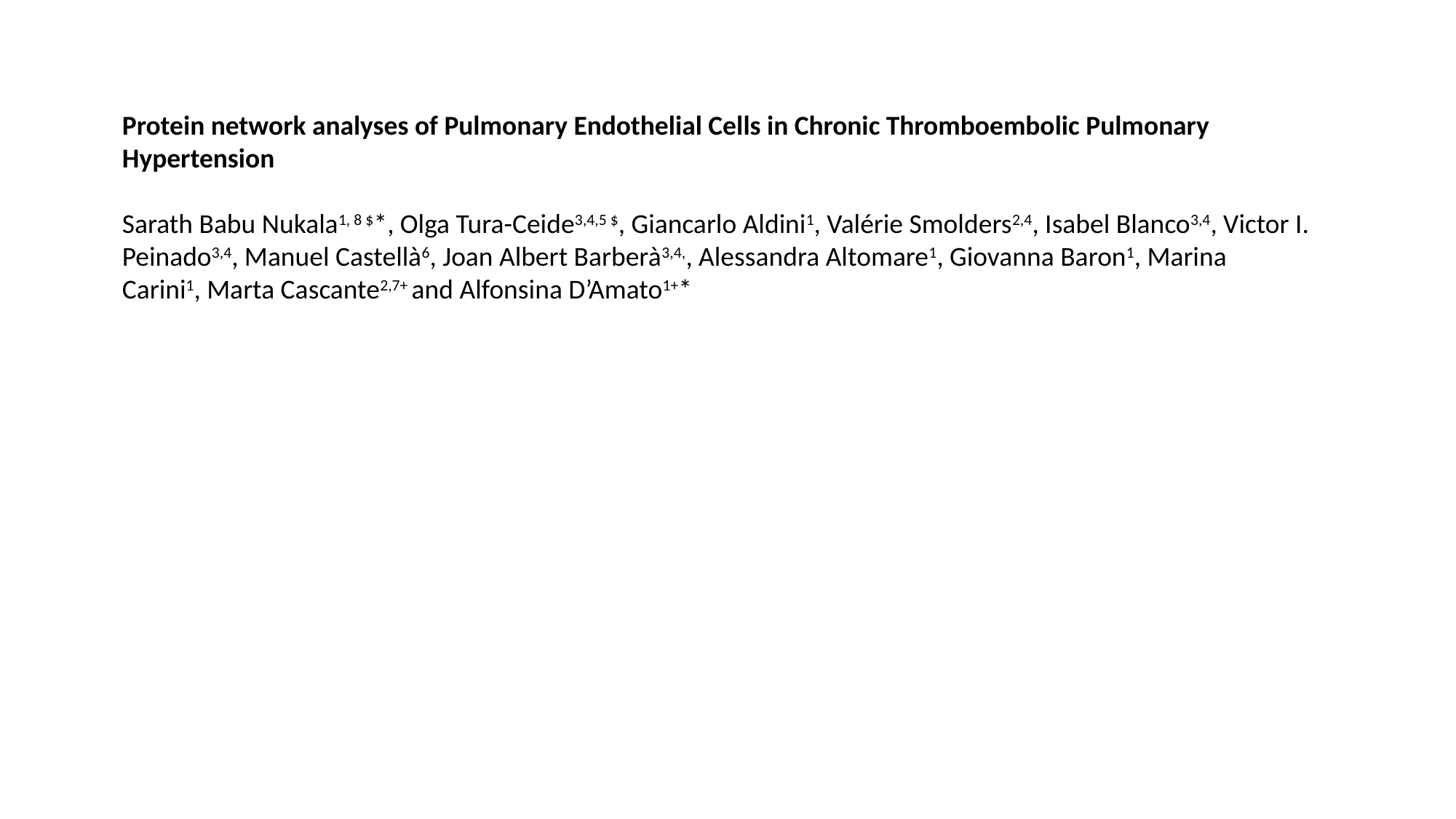

Protein network analyses of Pulmonary Endothelial Cells in Chronic Thromboembolic Pulmonary Hypertension
Sarath Babu Nukala1, 8 $*, Olga Tura-Ceide3,4,5 $, Giancarlo Aldini1, Valérie Smolders2,4, Isabel Blanco3,4, Victor I. Peinado3,4, Manuel Castellà6, Joan Albert Barberà3,4,, Alessandra Altomare1, Giovanna Baron1, Marina Carini1, Marta Cascante2,7+ and Alfonsina D’Amato1+*

## Slide 2
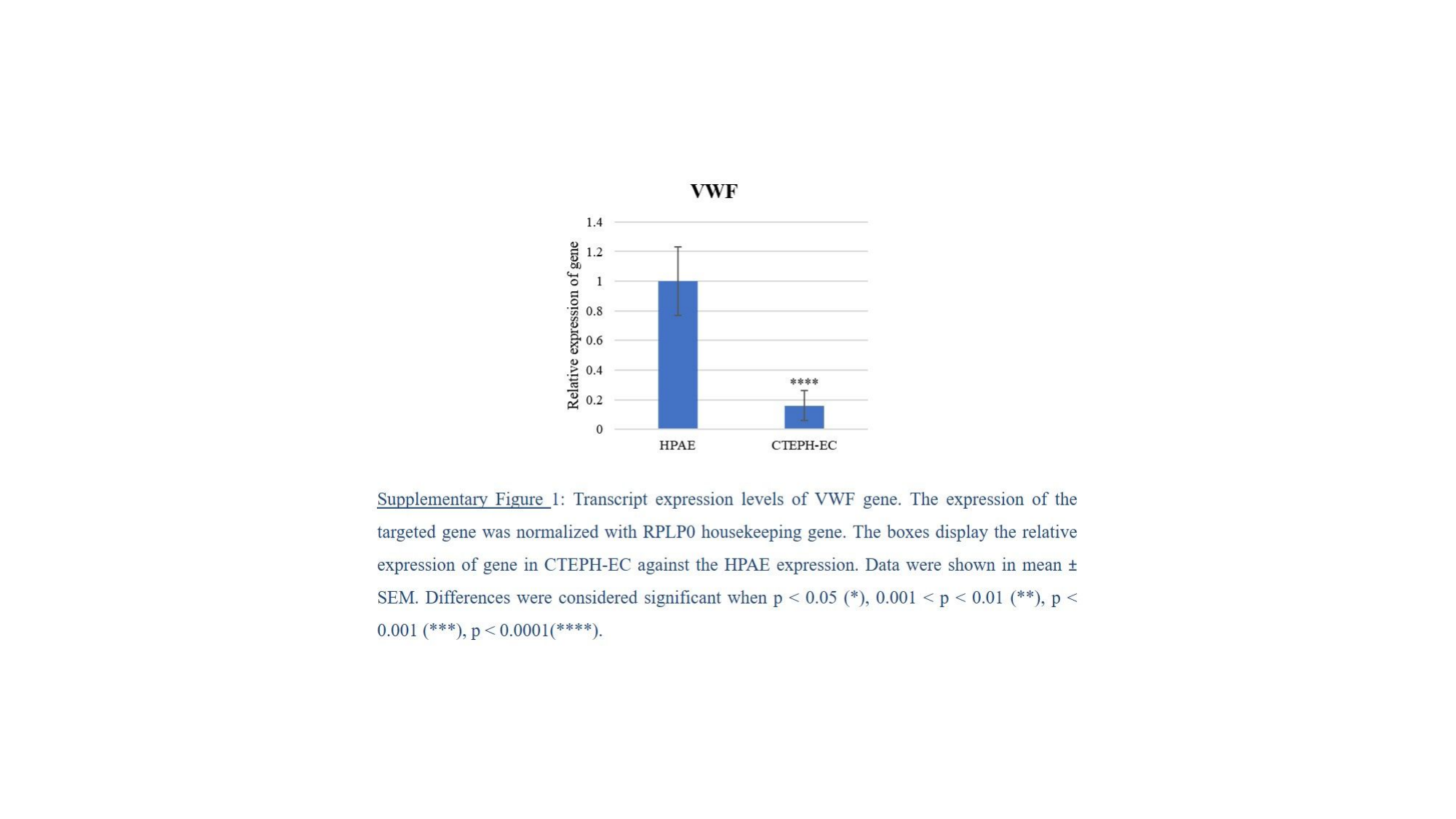

## Slide 3
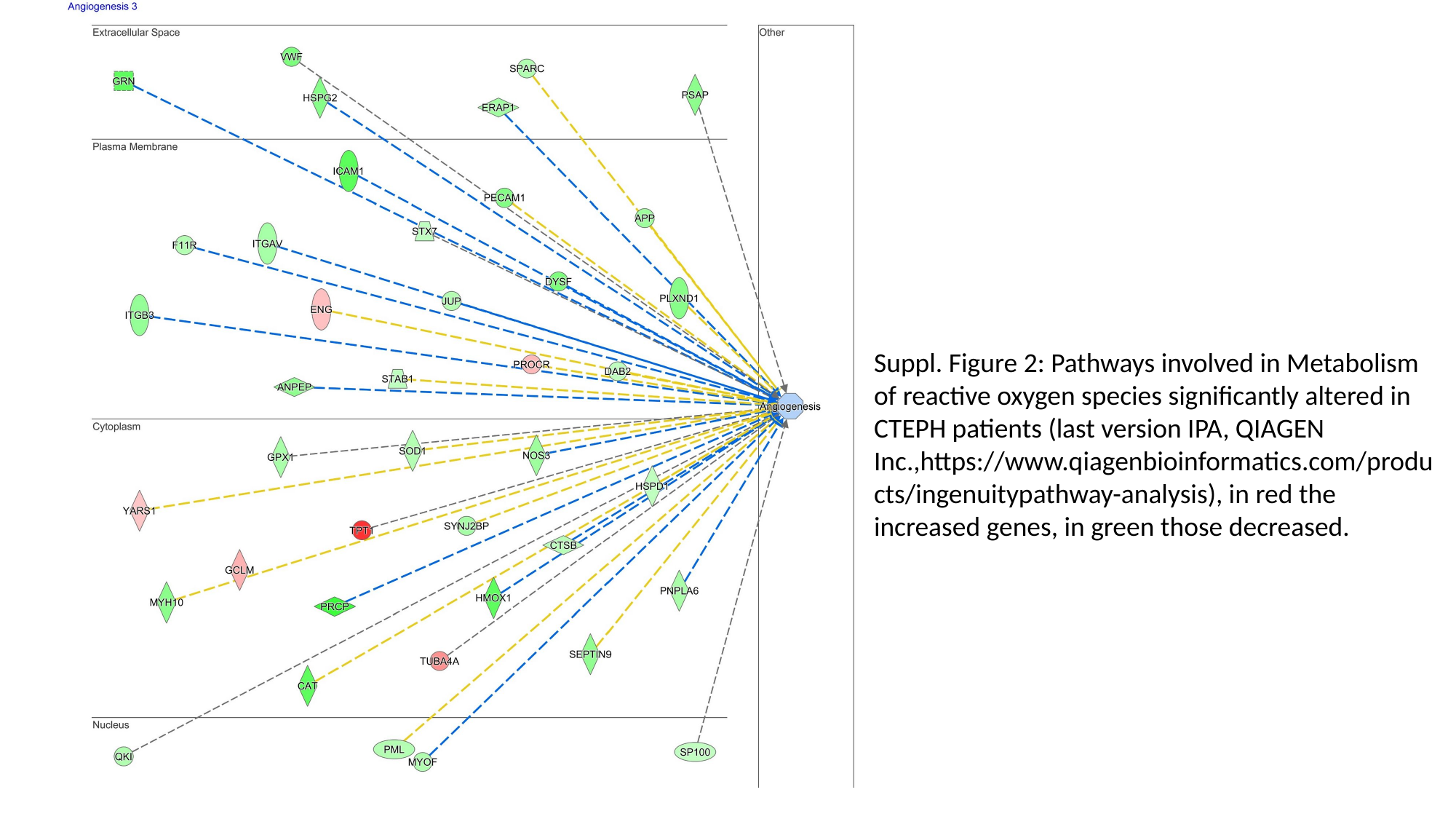

Suppl. Figure 2: Pathways involved in Metabolism of reactive oxygen species significantly altered in CTEPH patients (last version IPA, QIAGEN Inc.,https://www.qiagenbioinformatics.com/products/ingenuitypathway-analysis), in red the increased genes, in green those decreased.

## Slide 4
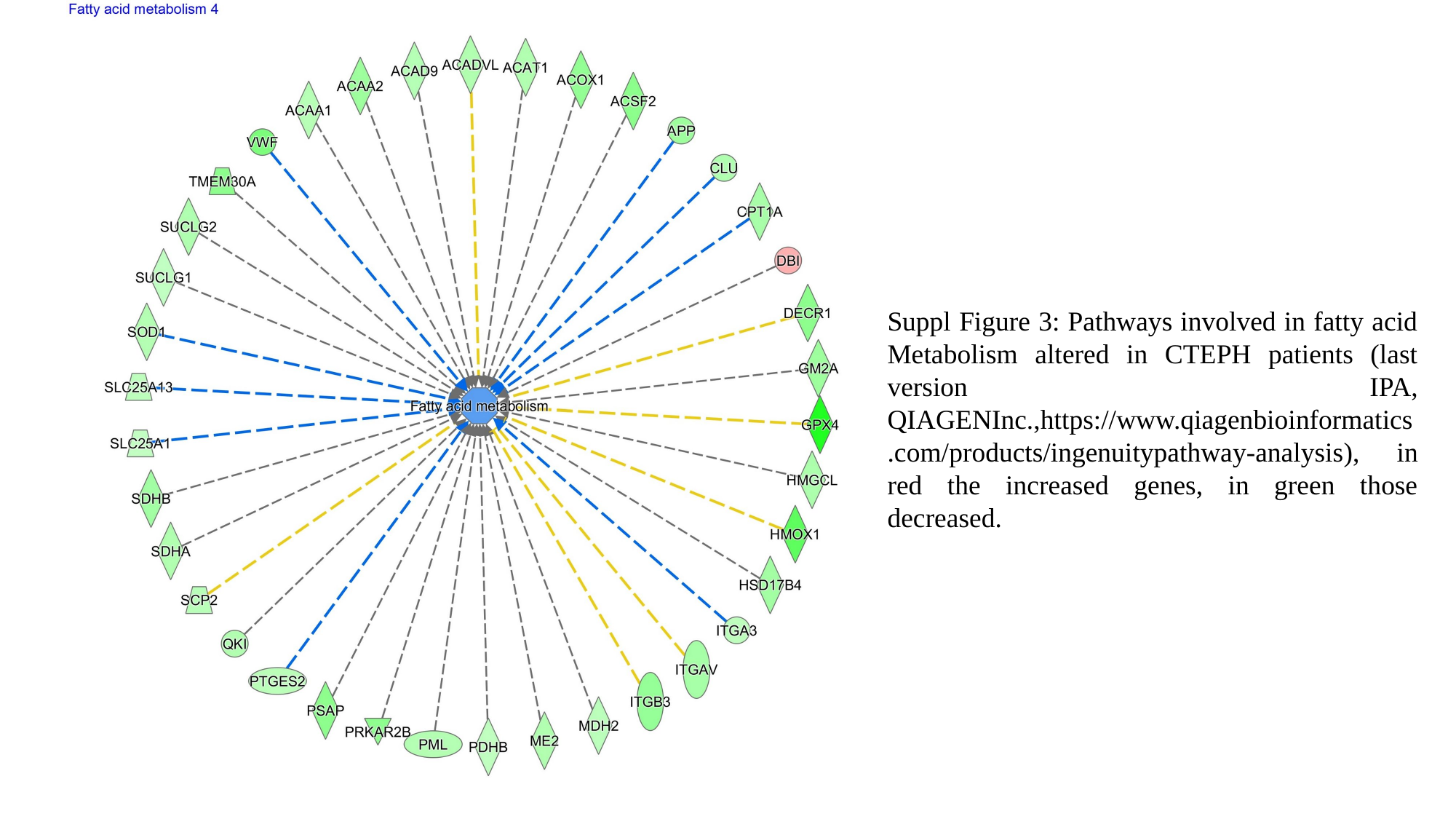

Suppl Figure 3: Pathways involved in fatty acid Metabolism altered in CTEPH patients (last version IPA, QIAGENInc.,https://www.qiagenbioinformatics.com/products/ingenuitypathway-analysis), in red the increased genes, in green those decreased.

## Slide 5
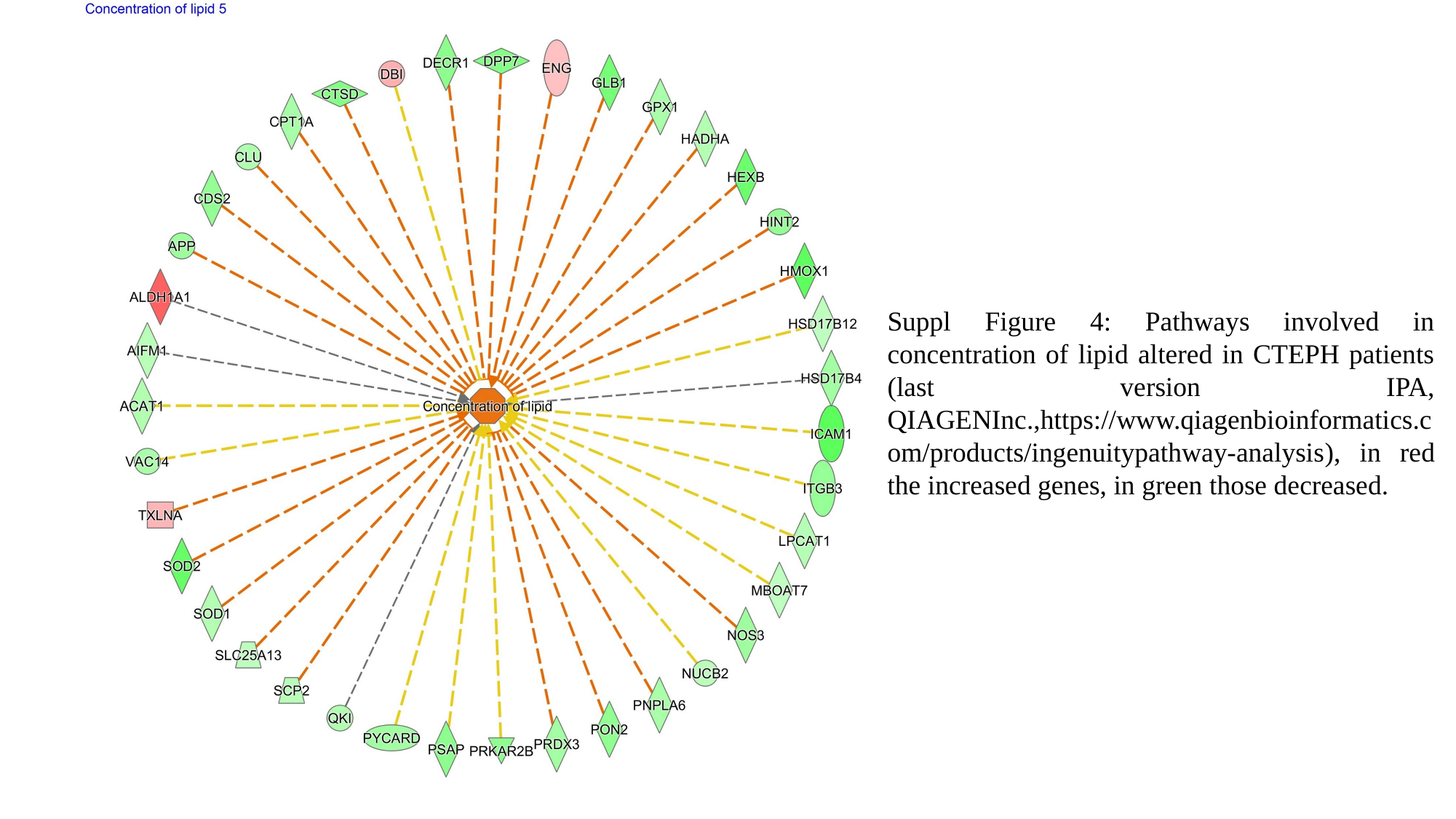

Suppl Figure 4: Pathways involved in concentration of lipid altered in CTEPH patients (last version IPA, QIAGENInc.,https://www.qiagenbioinformatics.com/products/ingenuitypathway-analysis), in red the increased genes, in green those decreased.

## Slide 6
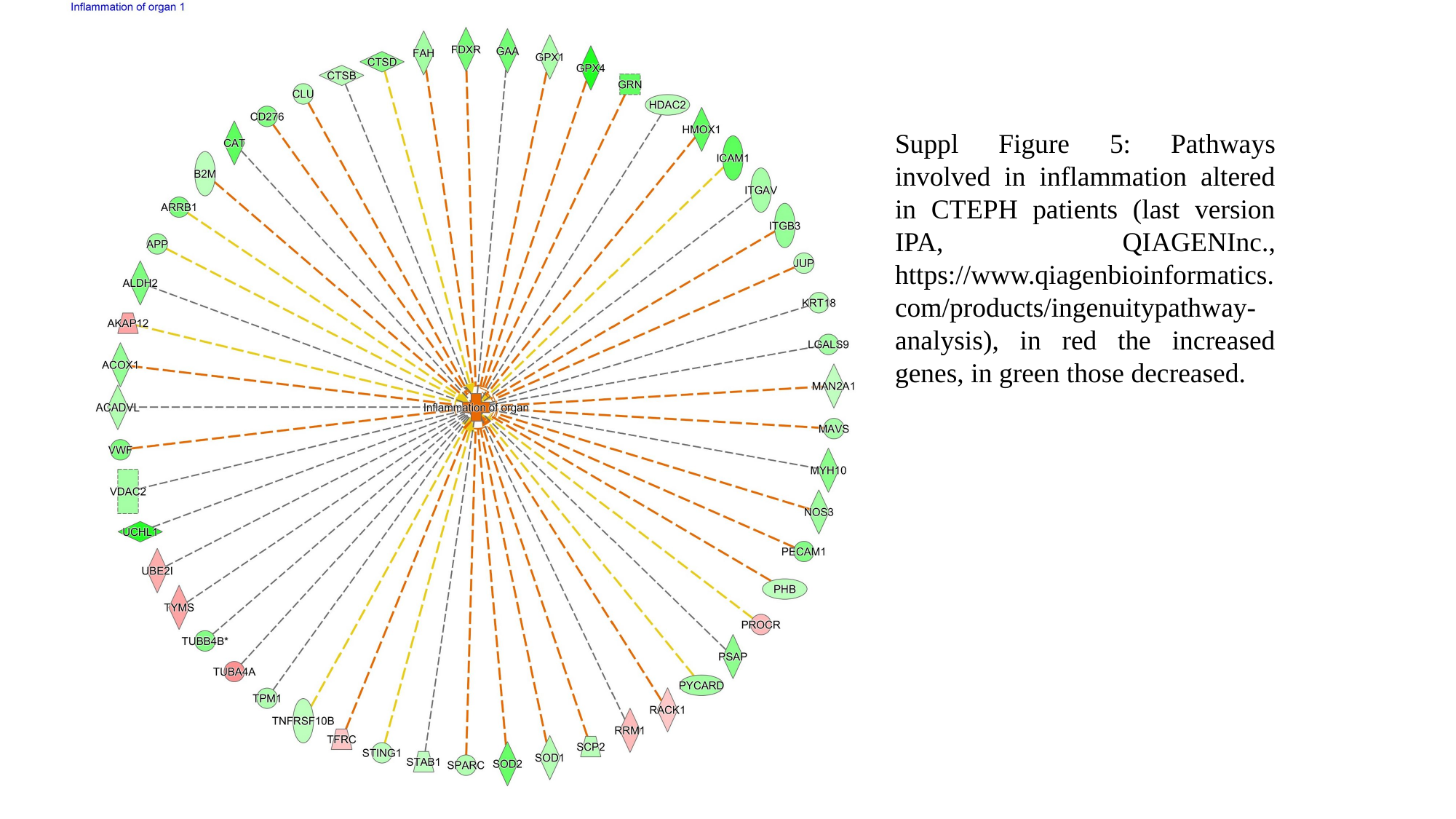

Suppl Figure 5: Pathways involved in inflammation altered in CTEPH patients (last version IPA, QIAGENInc., https://www.qiagenbioinformatics.com/products/ingenuitypathway-analysis), in red the increased genes, in green those decreased.
